# Supplementary material for: HIVprotI: an integrated web based platform for prediction and design of HIV proteins inhibitors
Source: J Cheminform. 2018 Mar 9;10:12. doi: 10.1186/s13321-018-0266-y (PMC5845081; doi:10.1186/s13321-018-0266-y)
Supplement: Supplementary file 2 — Additional file 2. Source code of HIVProtI web server. [file 13321_2018_266_MOESM2_ESM.zip › HIVprotI_Source-code/hivprotI/weka-3-6-6/doc/weka/classifiers/trees/ft/FTtree.html]

FTtree


---


|  |  |  |
| --- | --- | --- |
| **Overview** \| **Package** \| **Class** \| **Tree** \| **Deprecated** \| **Index** \| **Help** \| **Weka's home** | |  |
| **PREV CLASS**   NEXT CLASS | **FRAMES**    **NO FRAMES**     **All Classes** |  |
| SUMMARY: NESTED \| FIELD \| CONSTR \| METHOD | DETAIL: FIELD \| CONSTR \| METHOD |  |


---


## weka.classifiers.trees.ft Class FTtree

```
java.lang.Object
  weka.classifiers.Classifier
      weka.classifiers.trees.lmt.LogisticBase
          weka.classifiers.trees.ft.FTtree
```

**All Implemented Interfaces:**: java.io.Serializable, java.lang.Cloneable, CapabilitiesHandler, OptionHandler, RevisionHandler, WeightedInstancesHandler

**Direct Known Subclasses:**: FTInnerNode, FTLeavesNode, FTNode

---

``` public abstract class FTtree extends LogisticBase ```

Abstract class for Functional tree structure.

**Version:**
:   $Revision: 1.4 $

**Author:**
:   Jo\~{a}o Gama, Carlos Ferreira

**See Also:**: Serialized Form

---

**Constructor Summary**

`FTtree()`


| **Method Summary** | |
| --- | --- |
| `int` | `assignIDs(int lastID)`             Assigns unique IDs to all nodes in the tree |
| `int` | `assignLeafModelNumbers(int leafCounter)`             Assigns numbers to the logistic regression models at the leaves of the tree |
| `abstract  void` | `buildClassifier(Instances data)`             Method for building a Functional Tree (only called for the root node). |
| `abstract  void` | `buildTree(Instances data, SimpleLinearRegression[][] higherRegressions, double totalInstanceWeight, double higherNumParameters)`             Abstract method for building the tree structure. |
| `void` | `cleanup()`             Cleanup in order to save memory. |
| `abstract  double[]` | `distributionForInstance(Instance instance)`             Returns the class probabilities for an instance given by the Functional tree. |
| `int` | `getConstError(double[] probsConst)` |
| `java.lang.String` | `getModelParameters()`             Returns a string describing the number of LogitBoost iterations performed at this node, the total number of LogitBoost iterations performed (including iterations at higher levels in the tree), and the number of training examples at this node. |
| `java.util.Vector` | `getNodes()`             Return a list of all inner nodes in the tree |
| `void` | `getNodes(java.util.Vector nodeList)`             Fills a list with all inner nodes in the tree |
| `int` | `getNumInnerNodes()`             Method to count the number of inner nodes in the tree |
| `int` | `getNumLeaves()`             Returns the number of leaves in the tree. |
| `java.lang.String` | `getRevision()`             Returns the revision string. |
| `java.lang.String` | `graph()`             Returns graph describing the tree. |
| `boolean` | `hasModels()`             Returns true if the logistic regression model at this node has changed compared to the one at the parent node. |
| `double[]` | `modelDistributionForInstance(Instance instance)`             Returns the class probabilities for an instance according to the logistic model at the node. |
| `java.lang.String` | `modelsToString()`             Returns a string describing the logistic regression function at the node. |
| `int` | `numLeaves()`             Returns the number of leaves (normal count). |
| `int` | `numNodes()`             Returns the number of nodes. |
| `abstract  double` | `prune()`             Abstract Method that prunes a tree using C4.5 pruning procedure. |
| `java.lang.String` | `toString()`             Returns a description of the Functional tree (tree structure and logistic models) |

**Methods inherited from class weka.classifiers.trees.lmt.LogisticBase**

`getMaxIterations, getNumRegressions, getUseAIC, getUsedAttributes, getWeightTrimBeta, percentAttributesUsed, setHeuristicStop, setMaxIterations, setUseAIC, setWeightTrimBeta`

**Methods inherited from class weka.classifiers.Classifier**

`classifyInstance, debugTipText, forName, getCapabilities, getDebug, getOptions, listOptions, makeCopies, makeCopy, setDebug, setOptions`

**Methods inherited from class java.lang.Object**

`equals, getClass, hashCode, notify, notifyAll, wait, wait, wait`

**Constructor Detail**

### FTtree

```
public FTtree()
```


**Method Detail**

### buildClassifier

```
public abstract void buildClassifier(Instances data)
                              throws java.lang.Exception
```

:   Method for building a Functional Tree (only called for the root node).
    Grows an initial Functional Tree.

    :   **Overrides:**: `buildClassifier` in class `LogisticBase`
    :   **Parameters:**: `data` - the data to train with **Throws:**: `java.lang.Exception` - if something goes wrong

---


### buildTree

```
public abstract void buildTree(Instances data,
                               SimpleLinearRegression[][] higherRegressions,
                               double totalInstanceWeight,
                               double higherNumParameters)
                        throws java.lang.Exception
```

:   Abstract method for building the tree structure.
    Builds a logistic model, splits the node and recursively builds tree for child nodes.

    :   **Parameters:**: `data` - the training data passed on to this node: `higherRegressions` - An array of regression functions produced by LogitBoost at higher levels in the tree. They represent a logistic regression model that is refined locally at this node.: `totalInstanceWeight` - the total number of training examples: `higherNumParameters` - effective number of parameters in the logistic regression model built in parent nodes **Throws:**: `java.lang.Exception` - if something goes wrong

---


### prune

```
public abstract double prune()
                      throws java.lang.Exception
```

:   Abstract Method that prunes a tree using C4.5 pruning procedure.

    :   **Throws:**: `java.lang.Exception` - if something goes wrong

---


### getNumInnerNodes

```
public int getNumInnerNodes()
```

:   Method to count the number of inner nodes in the tree

    :   **Returns:**: the number of inner nodes

---


### getNumLeaves

```
public int getNumLeaves()
```

:   Returns the number of leaves in the tree.
    Leaves are only counted if their logistic model has changed compared to the one of the parent node.

    :   **Returns:**: the number of leaves

---


### getNodes

```
public java.util.Vector getNodes()
```

:   Return a list of all inner nodes in the tree

    :   **Returns:**: the list of nodes

---


### getNodes

```
public void getNodes(java.util.Vector nodeList)
```

:   Fills a list with all inner nodes in the tree

    :   **Parameters:**: `nodeList` - the list to be filled

---


### getConstError

```
public int getConstError(double[] probsConst)
```

:   **Type Parameters:**: `any` - probsConst

---


### hasModels

```
public boolean hasModels()
```

:   Returns true if the logistic regression model at this node has changed compared to the
    one at the parent node.

    :   **Returns:**: whether it has changed

---


### modelDistributionForInstance

```
public double[] modelDistributionForInstance(Instance instance)
                                      throws java.lang.Exception
```

:   Returns the class probabilities for an instance according to the logistic model at the node.

    :   **Parameters:**: `instance` - the instance **Returns:**: the array of probabilities **Throws:**: `java.lang.Exception`

---


### distributionForInstance

```
public abstract double[] distributionForInstance(Instance instance)
                                          throws java.lang.Exception
```

:   Returns the class probabilities for an instance given by the Functional tree.

    :   **Overrides:**: `distributionForInstance` in class `LogisticBase`
    :   **Parameters:**: `instance` - the instance **Returns:**: the array of probabilities **Throws:**: `java.lang.Exception` - if distribution can't be computed successfully

---


### toString

```
public java.lang.String toString()
```

:   Returns a description of the Functional tree (tree structure and logistic models)

    :   **Overrides:**: `toString` in class `LogisticBase`
    :   **Returns:**: describing string

---


### numLeaves

```
public int numLeaves()
```

:   Returns the number of leaves (normal count).

    :   **Returns:**: the number of leaves

---


### numNodes

```
public int numNodes()
```

:   Returns the number of nodes.

    :   **Returns:**: the number of nodes

---


### getModelParameters

```
public java.lang.String getModelParameters()
```

:   Returns a string describing the number of LogitBoost iterations performed at this node, the total number
    of LogitBoost iterations performed (including iterations at higher levels in the tree), and the number
    of training examples at this node.

    :   **Returns:**: the describing string

---


### assignIDs

```
public int assignIDs(int lastID)
```

:   Assigns unique IDs to all nodes in the tree

---


### assignLeafModelNumbers

```
public int assignLeafModelNumbers(int leafCounter)
```

:   Assigns numbers to the logistic regression models at the leaves of the tree

---


### modelsToString

```
public java.lang.String modelsToString()
```

:   Returns a string describing the logistic regression function at the node.

---


### graph

```
public java.lang.String graph()
                       throws java.lang.Exception
```

:   Returns graph describing the tree.

    :   **Throws:**: `java.lang.Exception` - if something goes wrong

---


### cleanup

```
public void cleanup()
```

:   Cleanup in order to save memory.

    :   **Overrides:**: `cleanup` in class `LogisticBase`

---


### getRevision

```
public java.lang.String getRevision()
```

:   Returns the revision string.

    :   **Specified by:**: `getRevision` in interface `RevisionHandler` **Overrides:**: `getRevision` in class `LogisticBase`
    :   **Returns:**: the revision


---


|  |  |  |
| --- | --- | --- |
| **Overview** \| **Package** \| **Class** \| **Tree** \| **Deprecated** \| **Index** \| **Help** \| **Weka's home** | |  |
| **PREV CLASS**   NEXT CLASS | **FRAMES**    **NO FRAMES**     **All Classes** |  |
| SUMMARY: NESTED \| FIELD \| CONSTR \| METHOD | DETAIL: FIELD \| CONSTR \| METHOD |  |


---
